# Association between ALDH2 rs671 G>A polymorphism and gastric cancer susceptibility in Eastern Asia

**DOI:** 10.18632/oncotarget.22060

**Published:** 2017-10-19

**Authors:** You Jiang, Jun Zhang, Yuee Wu, Jian Wang, Liang Li

**Affiliations:** ^1^ Department of General Surgery, Hefei Second People's Hospital, Anhui Medical University, Hefei, Anhui, 230011, China; ^2^ Department of Electrocardiogram Diagnosis, The Second Affiliated Hospital, Anhui Medical University, Hefei, Anhui, 230060, China; ^3^ Department of Pathology, The First Affiliated Hospital, Anhui Medical University, Hefei, Anhui, 230022, China

**Keywords:** ALDH2, polymorphism, meta-analysis, gastric cancer, risk

## Abstract

To date, the relationship between the aldehyde dehydrogenases-2 (ALDH2) rs671 G>A (Glu504Lys) polymorphism and gastric cancer (GC) risk has not been thoroughly elucidated. To derive a more precise estimation of the effect of the ALDH2 rs671 G>A polymorphism on GC, we conducted this meta-analysis. We searched for qualified studies in the Embase, PubMed, Wang Fan and China National Knowledge Infrastructure databases. Pooled odds ratios (ORs) and 95% confidence intervals (CIs) were calculated to assess the association. A total of 6,421 GC patients and 8,832 control subjects were included in the present study. The pooled results indicated no significant relationship between the ALDH2 rs671 G>A polymorphism and GC susceptibility in all genetic models. A stratified analysis by country showed that the ALDH2 rs671 G>A polymorphism might be a risk factor for GC in Japan (Allele model: *P*_unadjusted_ = 0.034; Dominant model: *P*_unadjusted_ = 0.040); however, the result was nonsignificant when the Bonferroni correction and false discovery rate (FDR) were applied. In subgroup analyses by drinking status in the dominant model, our study revealed that the ALDH2 rs671 G>A polymorphism significantly increased the risk of GC for drinkers (dominant model: *P* < 0.001). No relationship between the ALDH2 rs671 G>A polymorphism and GC risk was observed in any other subgroup. Our present study indicated no association between the ALDH2 rs671 G>A polymorphism and GC risk in Eastern Asian populations. However, the ALDH2 rs671 G>A polymorphism can significantly increase GC risk for drinkers.

## INTRODUCTION

Gastric cancer (GC) is a frequent malignant tumour and is one of the primary causes of tumour-associated deaths in the world. In 2012, 951,600 patients were diagnosed with GC, accounting for 8% of cancer cases, and 723,100 patients died of GC, accounting for 10% of cancer deaths [1]. Recently, in many Western countries, the incidence of stomach cancer has gradually decreased. However, in Eastern Asia, the incidence and mortality rate of GC is rising, especially in China [2–4]. Gastric cancer has become a major public health problem, but the mechanism of carcinogenesis in gastric cancer is still unclear. It has been generally accepted that occurrence of stomach cancer is a multistep, complex and multifactorial process that involves diverse risk factors. Thus far, many environmental risk factors including drinking, smoking, *Helicobacter pylori* infection and micronutrient deficiency have been identified. Although people are exposed to the above factors, not all of them will develop GC, indicating that genetic factors are involved in the development of GC [5]. Single nucleotide polymorphisms (SNPs), the most frequent type of genetic mutations, may contribute to an individual's susceptibility to GC.

A SNP in the aldehyde dehydrogenase-2 (ALDH2) gene, rs671, is highly prevalent among the East Asian population and causes decreased ALDH2 enzyme activity which may result in an inability to eliminate acetaldehyde. Acetaldehyde, a metabolite of alcohol, is known to increase cancer susceptibility [6]. Polymorphisms of the human ALDH2 gene, which is located on chromosome 12q24, could alter blood acetaldehyde concentrations after alcohol intake [7]. The rs671 polymorphism (also called Glu487Lys) has been the most commonly investigated [8]. Several published studies that included East Asian populations have shown that the ALDH2 rs671 G>A polymorphism is associated with an increased risk of stomach cancer [9–11], and studies from Japan and Korea have indicated a possible interaction between alcohol intake and the ALDH2 rs671 polymorphism in the incidence of stomach cancerr [9,10]. ALDH2 polymorphism genotype frequencies are diverse among different ethnic populations [12]. The frequency of the ALDH2 rs671 A allele is very high in East Asians, and has not been observed in Africans, Caucasians, and Southeast Asians [13]. However, in East Asians, however, the frequency of the ALDH2 allele also varies among the Chinese, Koreans, and Japanese [14].

Previous case-control studies have reported a correlation between the ALDH2 rs671 G>A polymorphism and the development of stomach cancer, However, the results are still discrepant [9, 10, 15]. To clarify these findings, Cai et al. [16] conducted a meta- analysis of the associations between the ALDH2 rs671 G>A polymorphism and cancer. Their study showed a significantly increased risk for GC associated with the ALDH2 rs671 G>A polymorphism, but there were only three case-control studies analysing the relationship between the ALDH2 rs671 G>A polymorphism and stomach cancer in the stratified analysis. One meta-analysis by Wang et al. [17] also indicated an increased risk for GC associated with the ALDH2 rs671 A allele; however, they included just two studies in the subgroup analysis of their meta-analysis on ALDH2 rs671 G>A. Another meta-analysis conducted by Mocellin et al. [18] suggested the opposite result; there was no relationship between the ALDH2 rs671 G>A polymorphism and the occurrence of stomach cancer. The sample sizes of these three meta-analyses are extremely small and their results are conflicting. Therefore, to more accurately assess the correlation, we decided to conduct a meta-analysis on all eligible case-control studies. Furthermore, we performed several subgroup analyses stratified by country, source of controls, sex, smoking status and drinking status.

## RESULTS

### Characteristics of eligible studies

**Figure 1:**
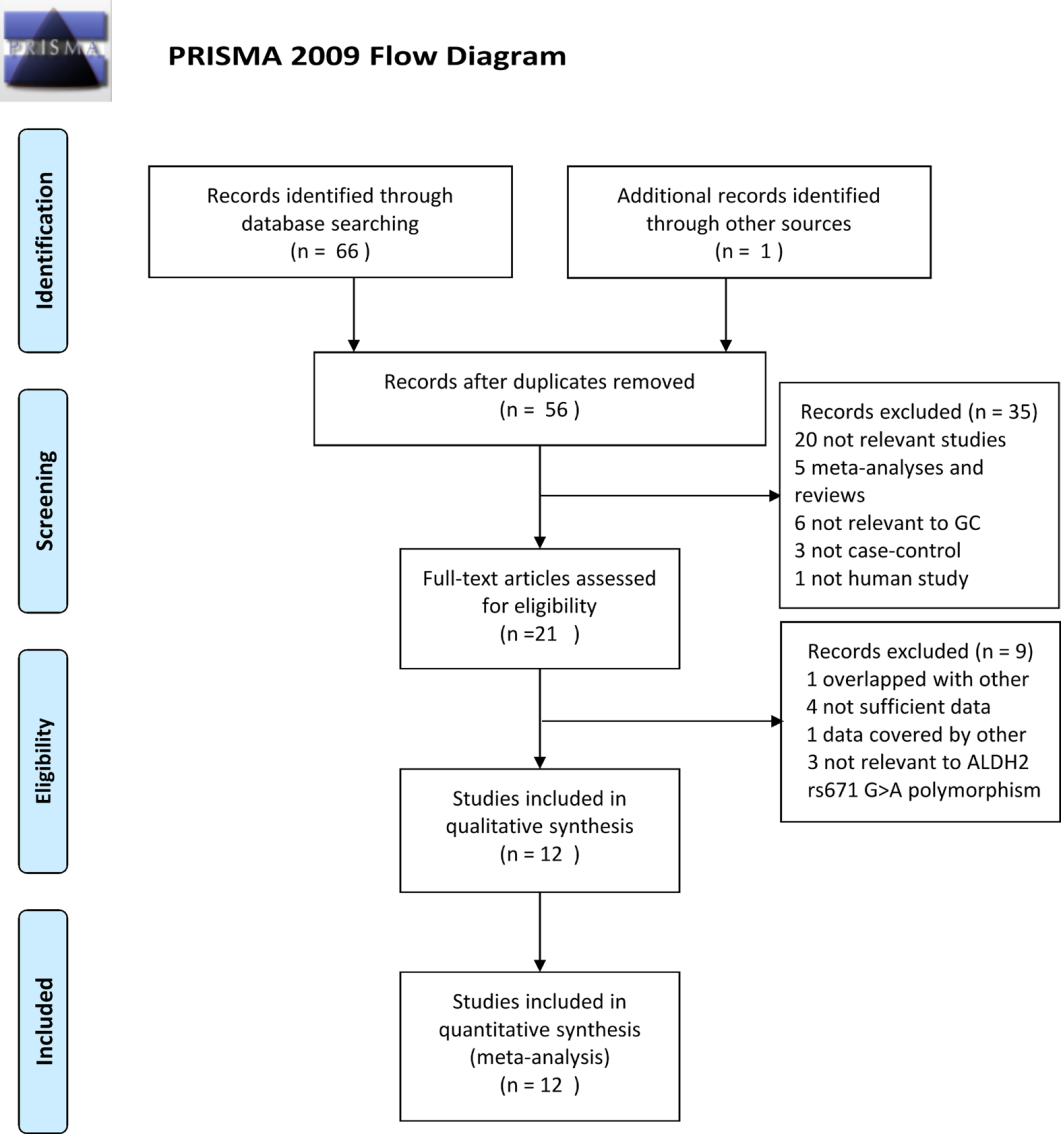
Flow chart of studies selection in this meta-analysis

Figure 1 shows a flow diagram of the study search process in our meta-analysis. The initial literature search identified 67 studies based on the selection strategy. Fifty-six studies were left after the removal of duplicate studies. Thirty-five studies were excluded after reviewing the titles and abstracts. Among these 35 studies, 20 were not relevant studies, 5 were meta-analyses or reviews, 6 were not relevant to GC, 3 were not case-control studies, and 1 was not a human study. The full text of the rest of the studies was reviewed, and 9 of these full-text studies were removed for the following reasons: 4 studies did not have sufficient data, 1 had data covered by another study, 1 had data that overlapped with another, and 3 were not relevant to the ALDH2 rs671 G>A polymorphism. Finally, 12 case-control studies about the ALDH2 rs671 G>A polymorphism and GC risk were eventually included for further analysis, encompassing a total of 6,421 cases and 8,832 control subjects [9, 10, 15, 19–27]. Table 1 shows the characteristics of eligible studies. All the cases were histologically confirmed in each of the included studies. All 12 case–control studies were performed with Asians and were published from 2009 to 2017. Among these studies, 8 were conducted in China, 2 were in Japan and 2 were in Korea. Six studies were hospital-based and six were population-based. All control subjects in the selected studies were within Hardy-Weinberg equilibrium (HWE), except for those in Yuan's study [21]. The relationship between the ALDH2 rs671 G>A polymorphism and GC risk in subgroups according to drinking status, sex and smoking status was assessed in 7, 2, and 2 studies, respectively. However, the author provided only the GG and GA + AA genotype counts of cases and controls without specific information on either the AA or GA genotype; thus, the dominant model was employed to assess the association between the ALDH2 rs671 G>A polymorphism and GC risk in the stratified analysis according to drinking status, sex and smoking status. The selected articles, assessed by the Newcastle-Ottawa Scale (NOS) score, ranged from 7 to 8 (Supplementary Table 1). The methodological quality of the included studies was reliable.

**Table 1:** Characteristics of eligible case-control studies included in this meta-analysis

First author	Year	Country	Ethnicity	Source of controls	Genotyping method	Number (case/control)	HWE	NOS score
Zhao et al. [19]	2014	China	Asians	HB	MALDI-TOFMS	308/308	Yes	7
Li et al. [20]	2009	China	Asians	PB	PCR-DHPLC	192/191	Yes	8
Zhou et al. [15]	2010	China	Asians	PB	PCR-DHPLC	201/199	Yes	8
Yuan et al. [21]	2016	China	Asians	PB	PCR-DHPLC	161/161	No	8
Hidaka et al. [22]	2015	Japan	Asians	PB	TaqMan	457/457	Yes	8
Yang et al. [23]	2016	Korea	Asians	HB	MassARRAY	450/1050	Yes	7
Cao et al. [24]	2010	China	Asians	PB	PCR-DHPLC	382/382	Yes	8
Chen et al. [25]	2016	China	Asians	HB	PCR-RFLP	246/274	Yes	7
Zhang et al. [26]	2017	China	Asians	HB	TaqMan	2686/3675	Yes	7
Matsuo et al. [10]	2013	Japan	Asians	HB	TaqMan	697/1372	Yes	7
Chang et al. [27]	2014	China	Asians	PB	PCR-RFLP	196/393	Yes	8
Shin et al. [9]	2011	Korea	Asians	HB	PCR-RFLP	445/370	Yes	8

PB: Population-based; HB: Hospital-based; HWE: Hardy-Weinberg equilibrium; NOS: Newcastle-Ottawa Scale; PCR-RFLP: polymerase chain reaction-restriction fragment length polymorphism; MALDI-TOF-MS: polymerase chain reaction–matrix-assisted laser desorption/ionization time-of-flight mass spectrometry; DHPLC: denaturing high performance liquid chromatography.

### Meta-analysis results

**Figure 2:**
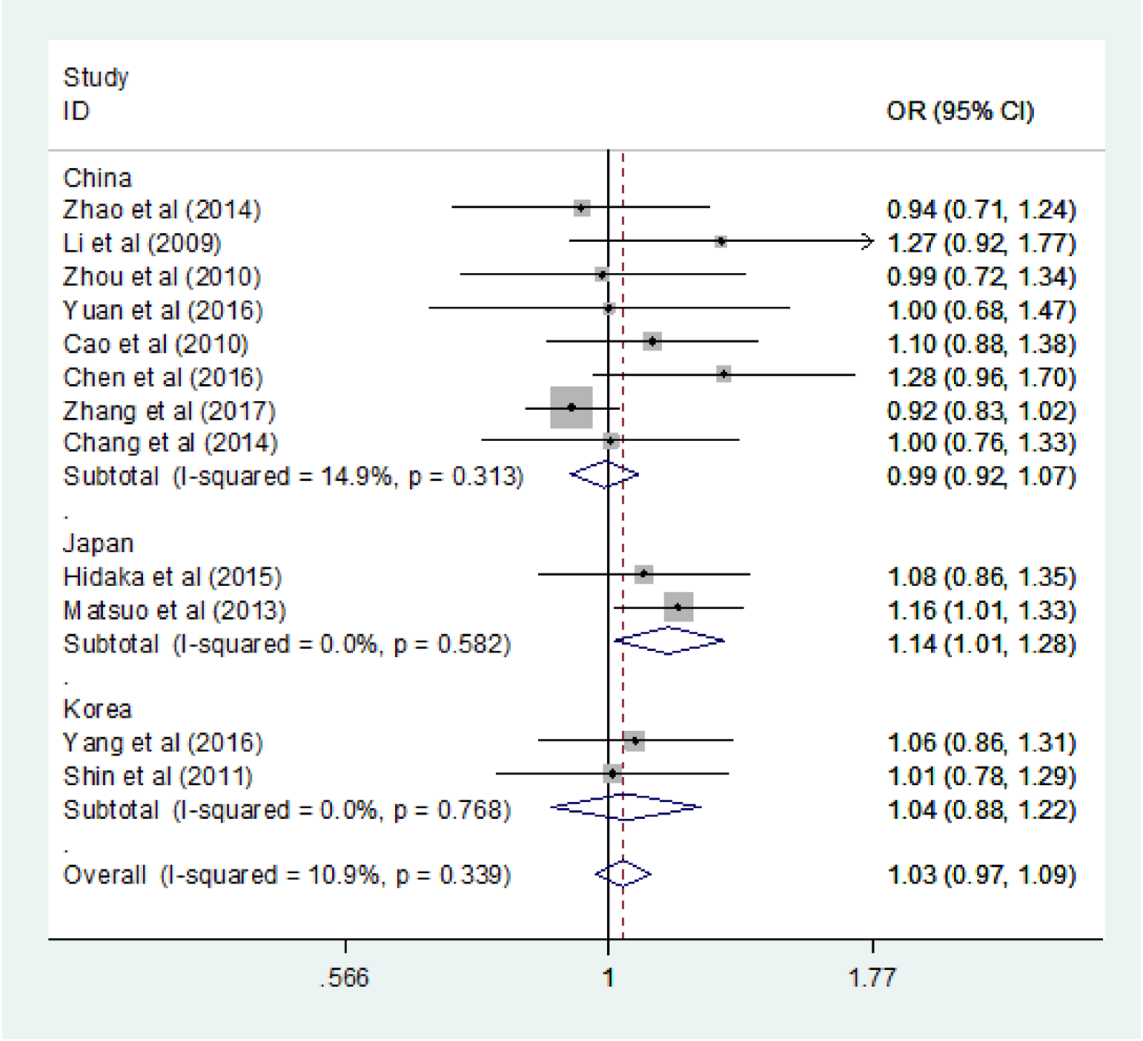
Forest plots of the ALDH2 rs671 G > A polymorphism and gastric cancer risk in subgroup by country (allele model: A vs. G)

**Figure 3:**
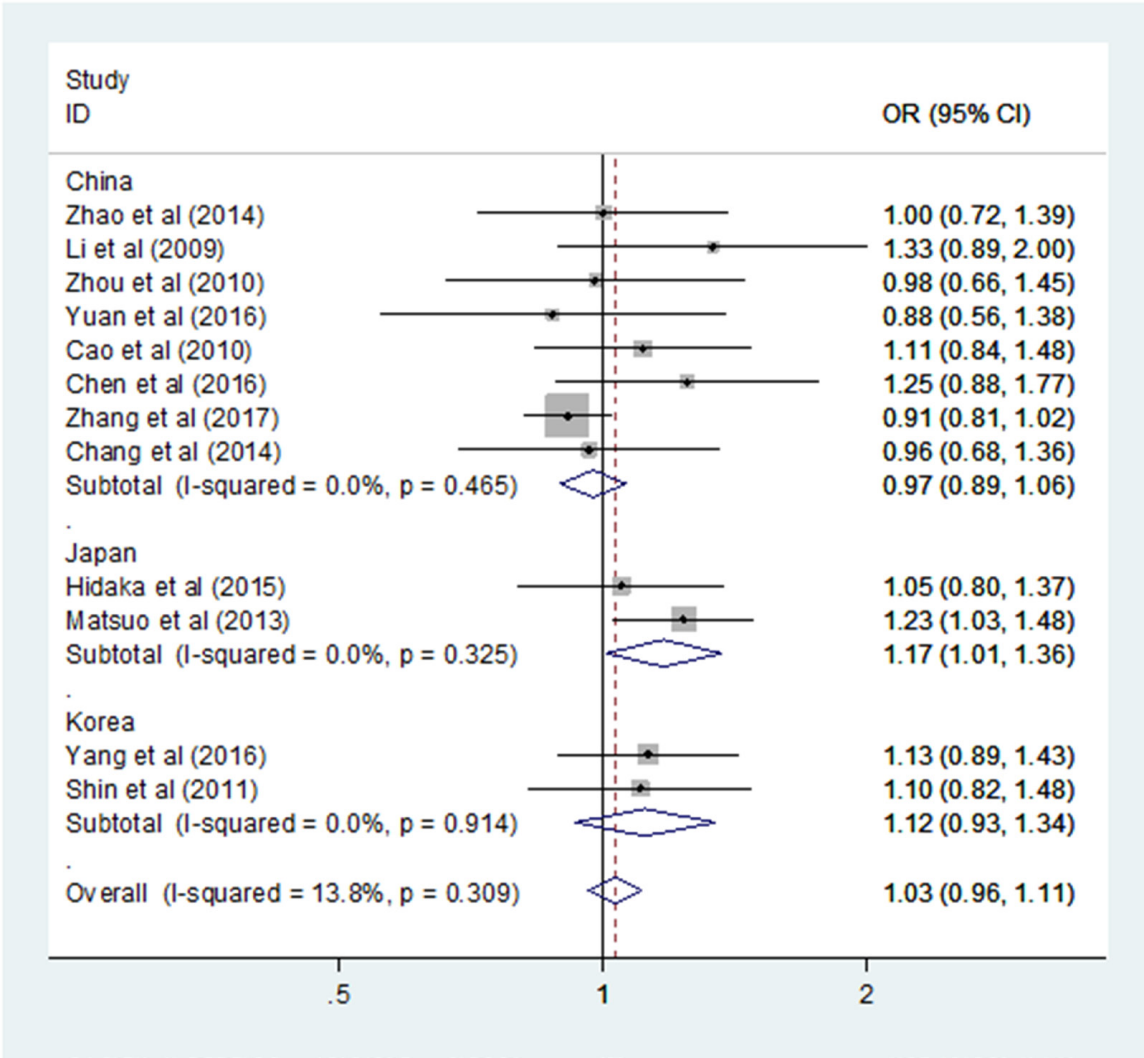
Forest plots of the ALDH2 rs671 G > A polymorphism and gastric cancer risk in subgroup by country (dominant model: GA + AA vs. GG)

**Figure 4:**
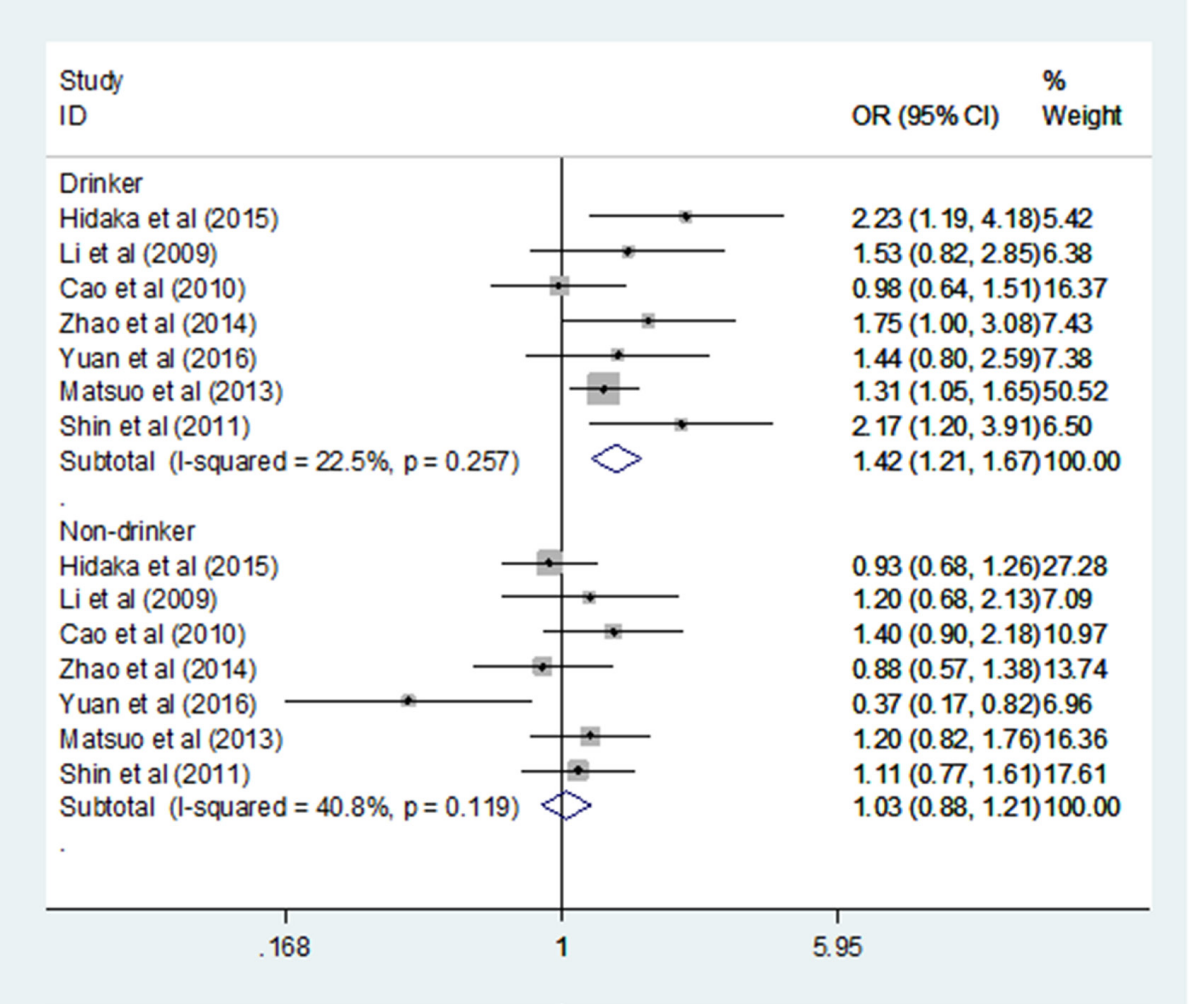
Forest plots of the ALDH2 rs671 G>A polymorphism and gastric cancer risk in the Drinker and Nonsmoker subgroup (dominant model: GA+AA vs. GG)

Table 2 shows the allele frequency and genotype distribution of the ALDH2 rs671 G > A polymorphism in cases and controls. Table 3 shows the main results of our meta-analysis, which contained a total of 12 case-control studies with 6,421 GC patients and 8,832 control subjects. Our meta-analysis revealed no significant association between the ALDH2 rs671 G > A polymorphism and GC risk in the overall pooled populations under any genetic model: AA VS GG (Odds ratio (OR) = 1.101, 95% confidence interval (CI) = 0.924–1.313, *I*^2^ = 23.2%, *P*_unadjusted_ = 0.282, *P*_Bonferroni_ = 1.000, *P*_FDR_ = 0.461); GA VS GG (OR = 1.030, 95% CI = 0.959–1.106, *I*^2^ = 12.7%, *P*_unadjusted_ = 0.423, *P*_Bonferroni_ = 1.000, *P*_FDR_ = 0.461); allele model (OR = 1.031, 95% CI = 0.972–1.093, *I*^2^ = 10.9%, *P*_unadjusted_ = 0.310, *P*_Bonferroni_ = 1.000, *P*_FDR_ = 0.461); recessive model (OR = 1.067, 95% CI = 0.898–1.268, *I*^2^ = 20.6%, *P*_unadjusted_ = 0.461, *P*_Bonferroni_ = 1.000, *P*_FDR_ = 0.461); and dominant model (OR = 1.033, 95% CI = 0.963–1.107, *I*^2^ = 13.8%, *P*_unadjusted_ = 0.365, *P*_Bonferroni_ = 1.000, *P*_FDR_ = 0.461). The heterogeneity of the included studies was low in all genetic models, and the fixed effect model was applied. The stratified analysis subgroup by country revealed an increased GC risk in Japan based on the allele model (OR = 1.138, 95% CI = 1.010–1.281, P = 0.034, *I*2 = 0.0%; Figure 2) and the dominant model (OR = 1.172, 95% CI = 1.008–1.364, P = 0.04, *I*2 = 0.0%; Figure 3), however, the result was not stable when the Bonferroni correction and false discovery rate (FDR) were applied (Allele model: *P*_Bonferroni_ = 0.170, *P*_FDR_ = 0.100; Dominant model: *P*_Bonferroni_ = 0.200, *P*_FDR_ = 0.100; Table 3). There was no statistically significant association between the ALDH2 rs671 G > A polymorphism and GC risk in China and Korea in any genetic model (Table 3). No correlation between the ALDH2 rs671 G > A polymorphism and susceptibility to GC was found in the hospital-based and population-based subgroups under any genetic model (Table 3). When sex, smoking status and drinking status subjects were analysed in the dominant model (GA+AA vs. GG), the ALDH2 rs671 G>A polymorphism significantly increased the risk of GC for drinkers (dominant model: OR = 1.421, 95% CI = 1.211–1.667, *P* < 0.001, *I^2^* = 22.5%; Figure 4), and there was no statistically significant association between the ALDH2 rs671 G > A polymorphism and GC in any other subgroup (Table 4 Supplementary Table 2).

**Table 2:** ALDH2 (rs671) polymorphisms genotype distribution and allele frequency in cases and controls

First author	Year	Genotype(N)												MAF	HWE
		Case				Control				Case		Control			
		Total	GG	GA	AA	Total	GG	GA	AA	G	A	G	A		
Zhao et al. [18]	2014	308	194	106	8	308	194	100	14	494	122	488	128	0.21	0.81
Li et al. [19]	2009	192	101	76	15	191	114	66	11	278	106	294	88	0.48	0.72
Zhou et al. [15]	2010	201	99	91	11	199	97	91	11	289	113	285	113	0.28	0.08
Yuan et al. [20]	2016	161	104	50	7	161	99	60	2	258	64	258	64	0.19	0.03
Hidaka et al. [21]	2015	457	287	149	21	457	292	150	15	723	191	734	180	0.20	0.42
Yang et al. [22]	2016	450	304	141	5	1050	736	292	22	749	151	1764	336	0.23	0.26
Cao et al. [23]	2010	382	196	161	25	382	206	155	21	553	211	567	197	0.26	0.24
Chen et al. [24]	2016	246	133	95	18	274	163	101	10	361	131	427	121	0.22	0.24
Zhang et al. [25]	2017	2686	1995	643	48	3675	2663	941	71	4633	739	6267	1083	0.15	0.25
Matsuo et al. [10]	2013	696	310	323	63	1372	683	580	109	943	449	1946	798	0.29	0.36
Chang et al. [26]	2014	196	108	76	12	393	213	160	20	292	100	586	200	0.25	0.15
Shin et al. [9]	2011	445	291	141	13	370	250	102	18	723	167	602	138	0.19	0.08

MAF: minor allele frequency; HWE: Hardy-Weinberg equilibrium.

**Table 3:** Meta-analysis results

Genetic model	Category	OR (95% CI)	*P*	Bon	FDR	Heterogeneity		Begg's test	Egger test
						*I*^2^	*P*	*P*	*P*
AA VS GG	Overall	1.101 [0.924; 1.313]	0.282	1.000	0.461	23.2%	0.215	0.837	0.872
	China	1.110 [0.879; 1.403]	0.380	1.000	0.693	20.4%	0.267		
	Japan	1.303 [0.963; 1.763]	0.087	0.435	0.109	0.0%	0.773		
	Korea	0.592 [0.329; 1.064]	0.080	0.400	0.155	0.0%	0.847		
	HB	0.592 [0.329; 1.064]	0.926	1.000	0.926	52.4%	0.062		
	PB	0.592 [0.329; 1.064]	0.074	0.370	0.220	0.0%	0.845		
GA VS GG	Overall	1.030 [0.959; 1.106]	0.423	1.000	0.461	12.7%	0.321	0.537	0.240
	China	0.965 [0.883; 1.055]	0.431	1.000	0.693	0.0%	0.611		
	Japan	1.152 [0.985; 1.350]	0.076	0.380	0.109	20.8%	0.261		
	Korea	1.176 [0.973; 1.422]	0.093	0.465	0.155	0.0%	0.937		
	HB	1.033 [0.952; 1.122]	0.435	1.000	0.926	48.6%	0.083		
	PB	1.019 [0.882; 1.176]	0.801	1.000	0.801	0.0%	0.726		
Allele model	Overall	1.031 [0.972; 1.093]	0.310	1.000	0.461	10.9%	0.339	0.945	0.185
	China	0.991 [0.921; 1.067]	0.815	1.000	0.815	14.9%	0.313		
	Japan	1.138 [1.010; 1.281]	0.034	0.170	0.100	0.0%	0.582		
	Korea	1.037 [0.883; 1.218]	0.659	1.000	0.659	0.0%	0.768		
	HB	1.016 [0.949; 1.088]	0.647	1.000	0.926	49.9%	0.076		
	PB	1.072 [0.957; 1.200]	0.229	1.000	0.381	0.0%	0.884		
Recessive model	Overall	1.067 [0.898; 1.268]	0.461	1.000	0.461	20.6%	0.241	0.745	0.813
	China	1.107 [0.878; 1.395]	0.389	1.000	0.693	15.6%	0.308		
	Japan	1.200 [0.897; 1.607]	0.220	1.000	0.220	0.0%	0.587		
	Korea	0.563 [0.314; 1.008]	0.053	0.265	0.155	0.0%	0.854		
	HB	0.973 [0.790; 1.198]	0.799	1.000	0.926	47.6%	0.089		
	PB	1.312 [0.960; 1.793]	0.088	0.440	0.220	0.0%	0.825		
Dominant model	Overall	1.033 [0.963; 1.107]	0.365	1.000	0.461	13.8%	0.309	0.537	0.163
	China	0.974 [0.894; 1.062]	0.554	1.000	0.693	0.0%	0.465		
	Japan	1.172 [1.008; 1.364]	0.040	0.200	0.100	0.0%	0.325		
	Korea	1.116 [0.928; 1.342]	0.365	1.000	0.456	0.0%	0.914		
	HB	1.026 [0.947; 1.112]	0.526	1.000	0.926	51.0%	0.070		
	PB	1.052 [0.916; 1.208]	0.476	1.000	0.595	0.0%	0.781		

OR: odds ratio; 95% CI: 95% confidence interval; Bon: *P*-value in Bonferroni testing; FDR: false discovery rate.

**Table 4:** Association between ALDH2 rs671 G>A polymorphism and sex, smoking status and drinking status of the gastric cancer patients based on dominant models

Subgroup analyses	Dominant model: GA + AA vs. GG						
	Heterogeneity						
	OR	95% CI	*P* value	*I*^2^	*P_Het_*	Effects model	No. of studies
Sex							
Male	1.049	0.942–1.168	0.385	93.9%	<0.001	R	2
Female	1.053	0.905–1.224	0.506	0.0%	0.980	F	2
Smoking status							
Smoker	1.212	0.851–1.728	0.286	0.0%	0.612	F	2
Nonsmoker	0.599	0.400–0.897	0.080	43.2%	0.184	F	2
Drinking status							
Drinker	1.421	1.211–1.667	**< 0.001**	22.5%	0.257	F	7
Nondrinker	1.031	0.879–1.209	0.707	40.8%	0.119	F	7

F: fixed effects model; R: random effects model.

### Sensitivity analyses

**Figure 5:**
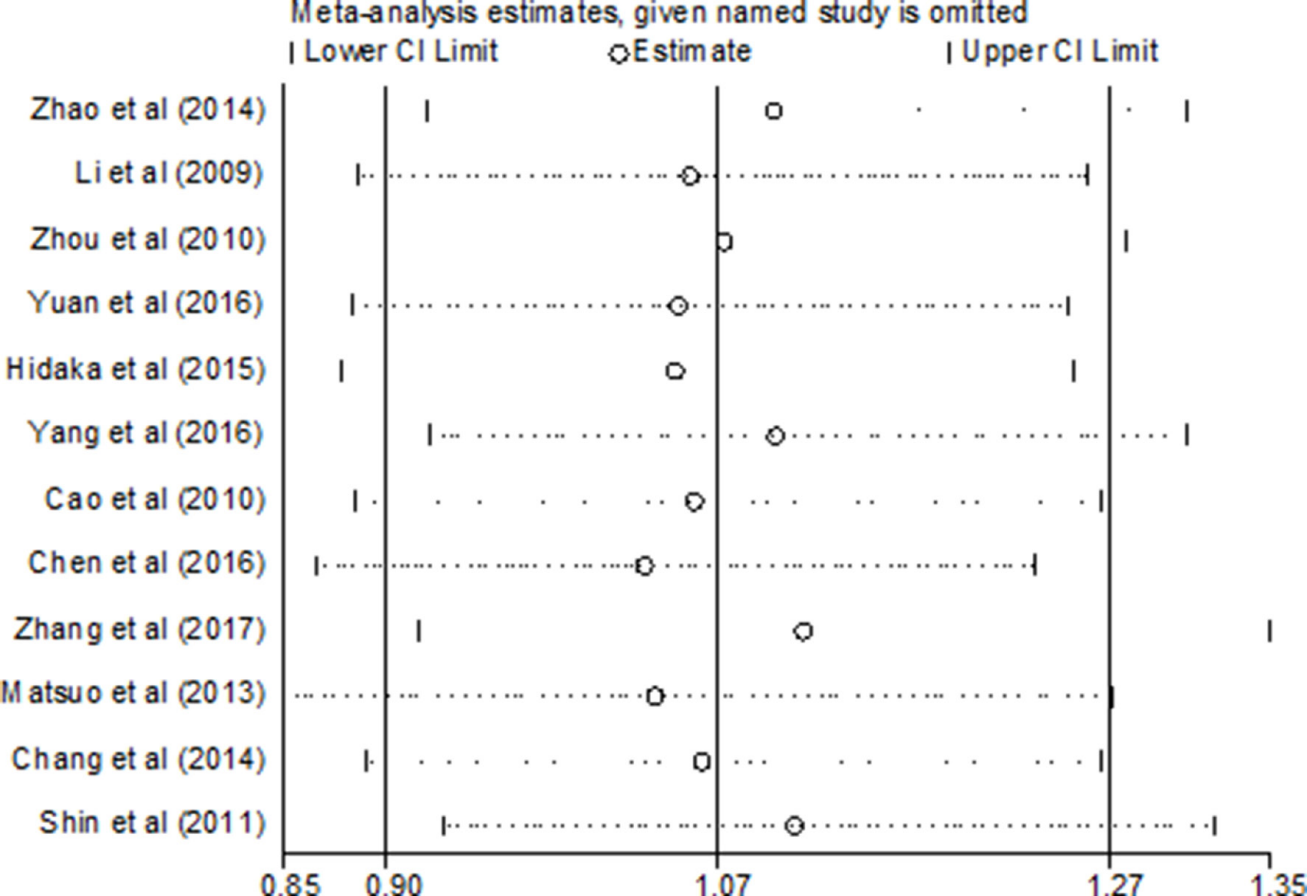
Sensitivity analysis of the ALDH2 rs671 G > A polymorphism and gastric cancer risk (recessive model: AA vs. GA + GG)

All the included studies were in accordance with HWE in their controls except Yuan’s, as shown in Table 1. To observe the impact of each single study on the pooled risk estimates, sensitivity analysis was performed with the leave-one-out cross-validation method. When removing any individual article from the analysis, the overall outcomes did not significantly change in the pooled ORs of the overall GC risk, indicating that our meta-analysis is credible and reliable (Figure 5, data not shown).

### Publication bias

**Figure 6:**
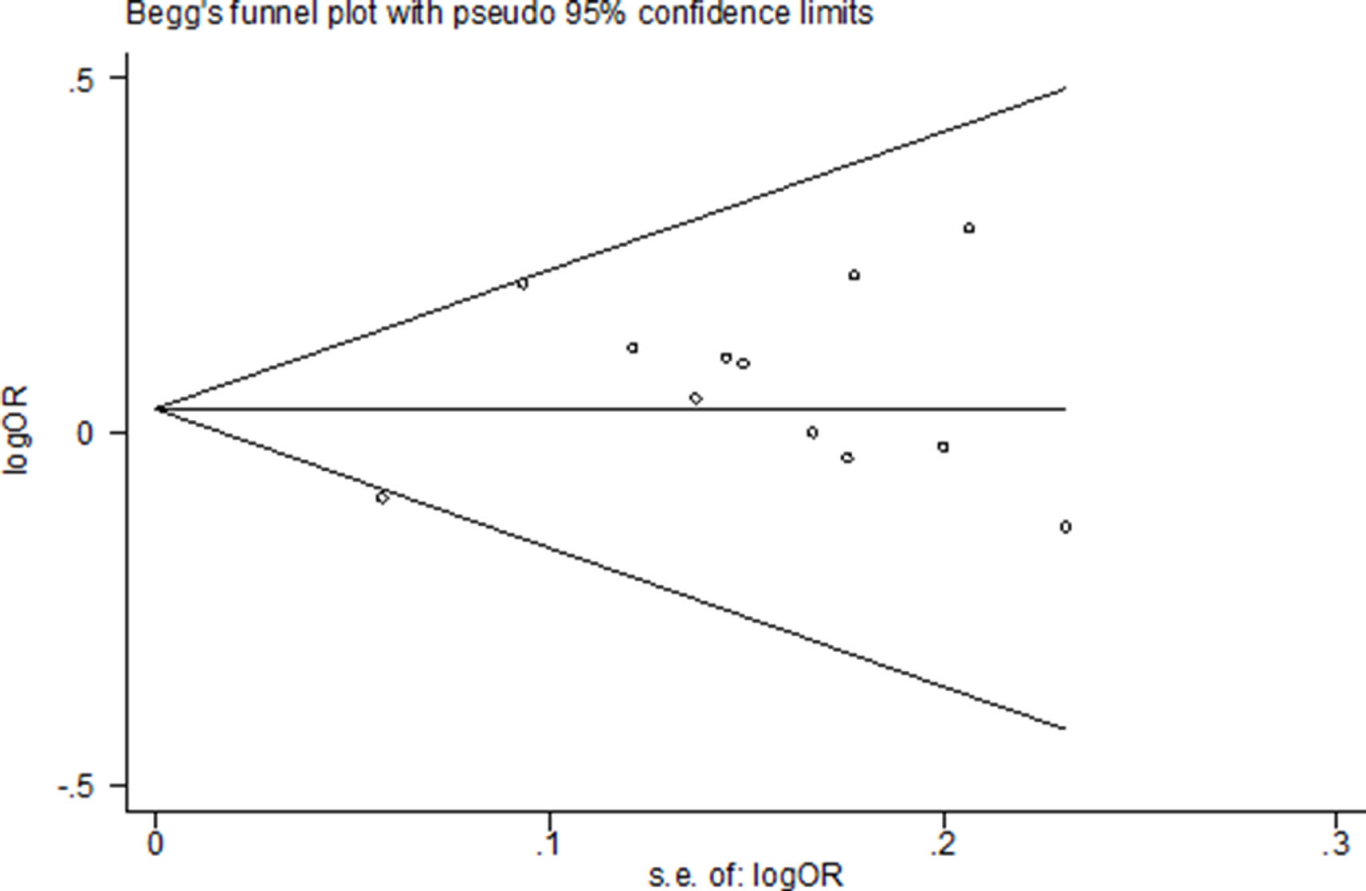
Funnel plot assessing evidence of publication bias from 12 studies (dominant model: GA+AA vs. GG)

Begg's funnel plot and Egger's test were used to evaluate publishing bias. Figure 6 shows that the funnel plot demonstrated no apparent asymmetry, suggesting there is no significant publication bias in the overall population. In our present meta-analysis, there was no evidence of publication bias observed by Begg's test and Egger's test (Table 3, Figure 6).

## DISCUSSION

ALDH2 is the main enzyme responsible for the metabolism of acetaldehyde, which increases cancer risk [28]. Several mutations have been confirmed in the human ALDH2 gene, including rs671 G>A, rs16941667 C>T, rs968529 C>T and rs886205 T>C [16, 29]. Among these alleles, the ALDH2 gene rs671 G>A, namely, the Glu504Lys polymorphism, is the most widely investigated and potentially functional. Several genome- wide association (GWA) studies have demonstrated that the ALDH2 gene rs671 G>A polymorphism can significantly increase the risk of some tumours, including upper aerodigestive tract (UADT) cancer and oesophageal squamous cell carcinoma (OSCC) [30, 31]. In recent years, an accumulation of published studies have investigated the relationship between the ALDH2 rs671 G>A polymorphism and the risk of GC; however, the results are still conflicting [9, 10].Therefore, to make a more accurate assessment of the correlation, we decided to perform the present meta-analysis on all eligible case-control studies.

Our meta-analysis included a total of 12 case-control studies with 6,421 GC patients and 8,832 control subjects and showed that there was no significant association between the ALDH2 rs671 G>A polymorphism and GC risk in the overall pooled population under all five models. GWAS plays an important role in the procedures for the discovery of genetic mutations and the reliability of meta-analysis. However, there are no previous relevant GWAS investigating the relationship between the ALDH2 rs671 G>A polymorphism and the GC risk. Three previous meta-analyses assessed the association of the ALDH2 rs671 G>A polymorphism and GC susceptibility [16, 17, 18]. One meta-analysis performed in 2014 by Wang et al. [17] included two case-control studies with a total of 1,079 cases and 1,754 controls. Their study indicated that the ALDH2 rs671 G>A polymorphism significantly increased the risk of GC. Another meta-analysis carried out in 2015 by Cai et al. [16] included three case-control studies with a total of 1,523 cases and 2,124 controls in the subgroup analysis. Their results also indicated a statistically significant association between the ALDH2 rs671 G>A polymorphism and GC risk. Both results were inconsistent with the findings of our meta-analysis. These conflicting findings are likely the result of the sample sizes and diverse genetic backgrounds. The third meta-analysis conducted in 2015 by Mocellin et al. [18] showed no relationship between ALDH2 rs671 G>A polymorphism and GC risk. Although their results were consistent with our study, the sample size in their study was also extremely small, and the statistical power of their study was low. The results of our meta-analysis, which included more case-control studies, have more sufficient statistical power and are more reliable.

When stratified by country, the subgroup study indicated that the ALDH2 rs671 G>A polymorphism significantly increased the risk of GC in Japan based on the allele model and dominant model (Allele model: *P*_unadjusted_ = 0.034; Dominant model: *P*_unadjusted_ = 0.040). There was no statistically significant relationship in China and Korea under any genetic models. This result suggests that the differences between countries might be a potential source of heterogeneity for this relationship. It is hypothesized that differences between countries might reflect diversity in alleles and genotypes among diverse ethnic populations. However, this result should be interpreted prudently and confirmed by more case–control studies, as the sample size in the Japan subgroup is extremely small. When the Bonferroni correction and FDR were applied to adjust for multiple comparisons, the result was unreliable and showed no association between the ALDH2 rs671 G>A polymorphism and GC risk in Japan under any models. No statistically significant relationships were found in the population-based and hospital-based subgroup according to the source of controls. When the subgroups were split up by sex, smoking status and drinking status in the dominant model, our study revealed that the ALDH2 rs671 G>A polymorphism significantly increased the risk of GC for drinkers. The impact of the ALDH2 rs671 G>A polymorphism on alcohol induced carcinogenesis has been identified in published studies of cancers of the UADT, including head and neck cancers and OSCC [32, 33]. Several studies have reported the relationship between the ALDH2 rs671 G>A polymorphism and GC risk among alcoholics; however, the results are inconsistent. Cao et al. [24] showed that the ALDH2 rs671 G>A polymorphism and alcohol drinking may not play an important role in the occurrence of gastric cancer. However, more studies have demonstrated the opposite result and obtained a positive result [9, 10, 19, 22]. Our meta-analysis assessed the association of the ALDH2 rs671 G>A polymorphism, alcohol drinking and GC risk with seven case-control studies and showed that the risk of stomach cancer among drinkers was increased by 1.4-fold compared with that among non-drinkers. No significant association between the ALDH2 rs671 G>A polymorphism and stomach cancer risk was found in any other subgroups.

Although our study is the most up-to-date meta-analysis and included all eligible case-control studies until July 2017, similar to other studies, our study shares several flaws with other studies in the following aspects. First, although no obvious publication bias was shown in our study, some bias is unavoidable because only published studies were included. Some published studies in accordance with the conditions may not be included. Second, as a systematic summary of the data, our study did not demonstrate an association at the level of basic experiments. However, due to the finite number of selected studies and samples, the published data included in our study did not have a sufficiently large sample size for comprehensive analysis. Third, the selected papers in our study were mostly from the Chinese population. The number of case-control studies in certain stratified analyses was too small to acquire a reliable association. Finally, only the dominant model was applied to assess the relationship when stratifying by sex, smoking status and drinking status due to insufficient data. More original data from a large sample of multiple centres is needed to confirm the relationship between the ALDH2 rs671 G>A polymorphism and GC susceptibility.

In conclusion, our meta-analysis showed no association between the ALDH2 rs671 G>A polymorphism and GC risk in Eastern Asian populations. The ALDH2 rs671 G>A polymorphism and alcohol drinking had a synergistic interaction for GC risk. Data from a large sample of further investigations are still needed to confirm the roles of ALDH2 in GC and validate these associations.

## MATERIALS AND METHODS

### Search strategy

To screen out eligible studies, we searched relevant studies in the Embase, PubMed, Wang Fan and China National Knowledge Infrastructure (CNKI) databases. No language limitation was used in the search and the last search was updated on July 16, 2017. The following search key terms were used: (‘ALDH2’ or ‘aldehyde’ or ‘dehydrogenases-2’) and (‘gastric carcinoma’ or ‘gastric cancer’ or ‘stomach cancer’) and (‘polymorphisms’ or ‘genotype’ or ‘polymorphism’). We also studied the reference lists of the included studies and recent reviews.

### Inclusion and exclusion criteria

The eligible studies included in this meta-analysis must have provided the following information: (1) the relationship between the ALDH2 rs671 G>A polymorphism and GC risk; (2) a case–control study; (3) sufficient data for estimating an OR and the corresponding 95% CI; and (4) GC diagnoses and the sources of cases and controls were clearly described in the study. Studies were excluded for the following reasons: (1) duplicate data; (2) insufficient data; and (3) abstracts, reviews, comments and editorial letters. The largest published studies were selected if the same or overlapping data were used.

### Extracted information

According to the above inclusion criteria, two investigators independently extracted information from all collected studies. Discrepancies were solved by discussion among all investigators. We collected the following characteristics from each eligible study: the first author, year of publication, ethnicity, country, study design, source of cases, genotyping methods, number of cases and controls, minor allele frequency (MAF) in controls, matching variables, genotypes, source of the control group (population or hospital-based controls), evidence of HWE in the control group, and others.

### Quality assessment

Two investigators independently assessed the quality of the included studies using the NOS. The NOS was performed to assess the study quality based on the following aspects: selection, comparability, and exposure situation in case-control studies. Rating scores ranged from 0 to 9. Studies with a score higher than seven were considered to be of good quality (Supplementary Table 1).

### Statistical analysis

HWE in the controls was assessed for each study with a goodness-of-fit test (Chi square or Fisher's exact test). HWE was considered to be in significant disequilibrium when *P* was less than 0.05. We assessed the strength of the association between the ALDH2 rs671 G>A polymorphism and GC susceptibility using ORs with 95% CIs in the co-dominant model (AA vs. GG and GA vs. GG), recessive model (AA vs. GA+GG), dominant model (GA+AA vs. GG), and allele model (A vs. G). We conducted subgroup tests according to country, source of controls, sex, smoking status and drinking status. The significance of pooled ORs was tested using Z test. Differences were considered statistically significant when *P* was less than 0.05. The *I*^2^ test was used to explore the heterogeneity among eligible studies [34]. The random (DerSimonian-Laird method) effect model was used to calculate the pooled OR when the *I*^2^ value > 50% and was considered to represent significant statistical heterogeneity [35]. The fixed (Mantel-Haenszel method) effect model was used to measure the pooled OR when the *I*^2^ value < 50% and was considered to represent less heterogeneity [36]. To explore the influence of each included study, a sensitivity analysis was performed by excluding one study at a time and then examining the pooled OR by repeating the meta-analysis. Publication bias was assessed using Begg's rank correlation method and Egger's weighted regression method (*P* < 0.05 was considered statistically significant). Funnel plots were also used to illustrate the publication bias [37, 38]. All statistical analyses were performed using STATA 12.0 soft-ware (version 12.0; STATA Corp. College Station, TX, USA) using two-tailed *P*-values. To adjust for multiple comparisons, the Bonferroni correction method and FDR were applied. The power of the meta-analysis for each polymorphism to detect some effect size was estimated according to the method recommended by Hedges and Pigott with *P* < 0.05 considered as statistically significant [39].

## SUPPLEMENTARY MATERIALS TABLES


