# Supplementary material for: Association between ALDH2 rs671 G>A polymorphism and gastric cancer susceptibility in Eastern Asia
Source: Oncotarget. 2017 Oct 19;8(60):102401–12. doi: 10.18632/oncotarget.22060 (PMC5731965; doi:10.18632/oncotarget.22060)
Supplement: Supplementary file 1 [file oncotarget-08-102401-s001.pdf]

Association between ALDH2 rs671 G>A polymorphism and gastric cancer susceptibility in Eastern Asia

SUPPLEMENTARY MATERIALS

Supplementary Table 1: Methodological quality of the included studies according to the Newcastle-Ottawa Scale (NOS)

Study	Selection (score)				Comparability (score)	Exposure (score)			
	Adequate definition of patient case	Representativeness of patients cases	Selection of controls	Definition of control	Control for important factor or additional factor	Ascertainment of exposure	Same method of ascertainment for participants	Non-response Rate	Total Score
Chen et al. [25]	*	*	NA	*	**	*	*	NA	7
Yang et al. [23]	*	*	NA	*	**	*	*	NA	7
Hidaka et al. [22]	*	*	*	*	*	*	*	*	8
Zhang et al. [26]	*	*	NA	*	**	*	*	NA	7
Cao et al. [24]	*	*	*	*	**	*	*	NA	8
Matsuo et al. [10]	*	*	NA	*	**	*	*	NA	7
Chang et al. [27]	*	*	*	*	**	*	*	NA	8
Shin et al. [9]	*	*	NA	*	**	*	*	NA	8
Li et al. [20]	*	*	*	*	**	*	*	NA	8
Zhou et al. [15]	*	*	*	*	**	*	*	NA	8
Zhao et al. [19]	*	*	NA	*	**	*	*	NA	7
Yuan et al. [21]	*	*	*	*	**	*	*	NA	8

Supplementary Table 2: Methodological quality of the included studies according to the Newcastle-Ottawa Scale (NOS)

Study	Year	Case		Control		Variables
		GA+AA	GG	GA+AA	GG	
Hidaka et al.	2015	39	110	17	107	Drinker
Li et al.	2009	33	62	25	72	Drinker
Cao et al.	2010	58	146	57	141	Drinker
Zhao et al.	2014	45	123	23	110	Drinker
Yuan et al.	2016	45	66	28	59	Drinker
Matsuo et al.	2013	204	253	344	561	Drinker
Shin et al.	2011	57	175	17	113	Drinker
Hidaka et al.	2015	131	177	148	185	Non-drinker
Li et al.	2009	58	39	52	42	Non-drinker
Cao et al.	2010	128	50	119	65	Non-drinker
Zhao et al.	2014	68	71	91	84	Non-drinker
Yuan et al.	2016	12	38	34	40	Non-drinker
Matsuo et al.	2013	179	49	340	112	Non-drinker
Shin et al.	2011	97	116	103	137	Non-drinker
Zhang et al.	2017	519	1562	517	1349	Male
Yang et al.	2016	308	518	800	1828	Male
Zhang et al.	2017	172	433	495	1314	Female
Yang et al.	2016	122	297	932	2383	Female
Yuan et al.	2016	42	62	25	51	Smoker
Zhao et al.	2014	68	110	62	114	Smoker
Zhao et al.	2014	45	82	52	80	Non-smoker
Yuan et al.	2016	15	42	37	48	Non-smoker
